# Common variants upstream of *MLF1* at 3q25 and within *CPZ* at 4p16 associated with neuroblastoma

**DOI:** 10.1371/journal.pgen.1006787

**Published:** 2017-05-18

**Authors:** Lee D. McDaniel, Karina L. Conkrite, Xiao Chang, Mario Capasso, Zalman Vaksman, Derek A. Oldridge, Anna Zachariou, Millicent Horn, Maura Diamond, Cuiping Hou, Achille Iolascon, Hakon Hakonarson, Nazneen Rahman, Marcella Devoto, Sharon J. Diskin

**Affiliations:** 1 Division of Oncology, Children’s Hospital of Philadelphia, Philadelphia, PA, United States of America; 2 Center for Childhood Cancer Research, Children’s Hospital of Philadelphia, Philadelphia, PA, United States of America; 3 The Center for Applied Genomics, Children’s Hospital of Philadelphia, Philadelphia, PA, United States of America; 4 University of Naples Federico II, Naples, Italy; 5 Ceinge—Biotecnologie Avanzate, Naples, Italy; 6 IRCCS SDN, Istituto di Ricerca Diagnostica e Nucleare, Naples, Italy; 7 Department of Biomedical and Health Informatics, Children’s Hospital of Philadelphia, Philadelphia, PA, United States of America; 8 Medical Scientist Training Program, Perelman School of Medicine at the University of Pennsylvania, Philadelphia, PA, United States of America; 9 Division of Genetics and Epidemiology, Institute of Cancer Research, London, United Kingdom; 10 Department of Pediatrics, Perelman School of Medicine, University of Pennsylvania, Philadelphia, PA, United States of America; 11 Division of Genetics, The Children’s Hospital of Philadelphia, Philadelphia, PA, United States of America; 12 University of Rome “La Sapienza”, Department of Molecular Medicine, Rome, Italy; 13 Department of Biostatistics, Epidemiology and Informatics, Perelman School of Medicine, University of Pennsylvania, Philadelphia, PA, United States of America; 14 Abramson Family Cancer Research Institute, Perelman School of Medicine at the University of Pennsylvania, Philadelphia, PA, United States of America; Univeristy of Southern California, UNITED STATES

## Abstract

Neuroblastoma is a cancer of the developing sympathetic nervous system that most commonly presents in young children and accounts for approximately 12% of pediatric oncology deaths. Here, we report on a genome-wide association study (GWAS) in a discovery cohort or 2,101 cases and 4,202 controls of European ancestry. We identify two new association signals at 3q25 and 4p16 that replicated robustly in multiple independent cohorts comprising 1,163 cases and 4,396 controls (3q25: rs6441201 combined P = 1.2x10^-11^, Odds Ratio 1.23, 95% CI:1.16–1.31; 4p16: rs3796727 combined P = 1.26x10^-12^, Odds Ratio 1.30, 95% CI: 1.21–1.40). The 4p16 signal maps within the carboxypeptidase Z (*CPZ*) gene. The 3q25 signal resides within the arginine/serine-rich coiled-coil 1 (*RSRC1*) gene and upstream of the myeloid leukemia factor 1 (*MLF1*) gene. Increased expression of *MLF1* was observed in neuroblastoma cells homozygous for the rs6441201 risk allele (P = 0.02), and significant growth inhibition was observed upon depletion of *MLF1* (P < 0.0001) in neuroblastoma cells. Taken together, we show that common DNA variants within *CPZ* at 4p16 and upstream of *MLF1* at 3q25 influence neuroblastoma susceptibility and *MLF1* likely plays an important role in neuroblastoma tumorigenesis.

## Author summary

Neuroblastoma is an embryonal tumor of the developing sympathetic nervous system that accounts for 12% of childhood cancer deaths. Approximately 1–2% of cases are inherited in an autosomal dominant fashion. These familial cases often harbor germline mutations in *ALK* or *PHOX2B*. However, the vast majority of neuroblastomas appear to arise sporadically. We are studying sporadic neuroblastoma through an ongoing genome-wide association study (GWAS). To date, this effort has identified single nucleotide polymorphisms (SNPs) within or upstream of *CASC15* and *CASC14*, *BARD1*, *LMO1*, *DUSP12*, *HSD17B12*, *DDX4*/*IL31RA*, *HACE1*, *LIN28B*, and *TP53*, along with a common copy number variation (CNV) within *NBPF23* at chromosome 1q21.1, each being highly associated with neuroblastoma. Here, we report on genome-wide association study (GWAS) comprising 3,264 neuroblastoma patients and 8,598 control subjects. We identify two new association signals at 3q25 and 4p16 (3q25: rs6441201 combined P = 1.2x10^-11^, Odds Ratio 1.23, 95% CI:1.16–1.31; 4p16: rs3796727 combined P = 1.26x10^-12^, Odds Ratio 1.30, 95% CI: 1.21–1.40). The 3q25 signal resides upstream of the *MLF1* gene and the 4p16 signal maps to the *CPZ* gene. We further demonstrate that neuroblastoma cells homozygous for the risk allele at 3q25 express higher levels of *MLF1* and that silencing of *MLF1* in neuroblastoma cells results in significant growth inhibition.

## Introduction

Neuroblastoma is a cancer of the developing sympathetic nervous system that most commonly affects children under 5 years of age, with a median age at diagnosis of 17 months [1]. Approximately 50% of cases present with disseminated disease at the time of diagnosis, and despite intense multi-modal therapy, the survival rate for this high-risk subset remains less than 50% [1]. Somatically acquired segmental DNA copy number alterations, such as *MYCN* amplification and deletions of 1p and 11q, are associated with aggressive disease and poor survival [2]. However, recent whole genome and exome sequencing studies have revealed a relative paucity of somatic point mutations in neuroblastoma tumors [3–6].

In terms of the etiology of neuroblastoma, only 1–2% of patients present with a family history of disease; the vast majority of cases appear to arise sporadically. Familial neuroblastoma is largely explained by germline mutations in *ALK* [7, 8] or *PHOX2B* [9, 10]. To understand the genetic basis of *sporadic* neuroblastoma, we are performing a genome-wide association study (GWAS). To date, this effort has identified single nucleotide polymorphisms (SNPs) within or upstream of *CASC15* [11, 12] and *CASC14* [11], *BARD1* [13, 14], *LMO1* [15], *DUSP12* [16], *HSD17B12* [16], *DDX4*/*IL31RA* [16]*, HACE1 [17], LIN28B [17]*, and *TP53* [18], along with a common copy number variation (CNV) within *NBPF23* [19] at chromosome 1q21.1, each being highly associated with neuroblastoma. Importantly, several of the neuroblastoma susceptibility genes identified by GWAS have been shown to not only influence disease initiation, but also drive tumor aggressiveness and/or maintenance of the malignant phenotype [15, 17, 20–22].

Here, to identify additional germline variants and genes influencing neuroblastoma tumorigenesis, we imputed genotypes across the genome (see Methods) and performed a discovery GWAS of genotyped and imputed variants in a cohort of 2,101 neuroblastoma patients and 4,202 control subjects of European ancestry [17]. This effort refined previously reported susceptibility loci and identified two new association signals at 3q25 and 4p16 which were replicated in three independent cohorts comprising 1,163 cases and 4,396 controls. In addition, based on expression quantitative trait loci (eQTL) analysis and *in vitro* studies following manipulation of candidate genes in neuroblastoma cell lines, we demonstrate that the 3q25 signal likely targets the myeloid leukemia factor 1 (*MLF1*) gene in neuroblastoma, resulting in increased *MLF1* expression and promoting cell growth.

## Results

### Discovery GWAS based on individuals of European ancestry

**Fig 1:**
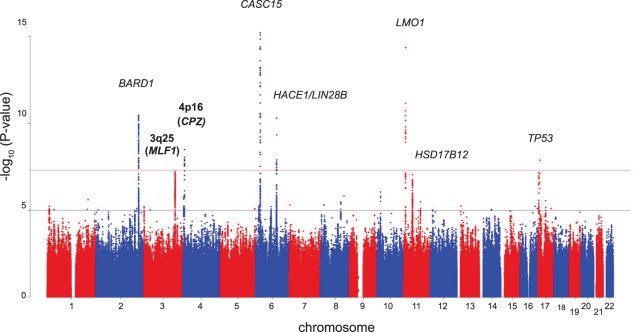
Manhattan plot of discovery results. Level of significance (-log_10_ transformed P values) for each SNP along the genome in chromosomal order is plotted. Red line: significance threshold of 5.0 x 10^−8^ considered for identification of novel loci. Previously identified susceptibility loci are labeled; new association signals identified at chromosomes 3q25 and 4p16 are indicated in bold.

To discover germline variants associated with neuroblastoma, we performed a GWAS following genome-wide genotype imputation in 2,101 neuroblastoma patients accrued through the North American-based Children’s Oncology Group (**S1 Table**) and 4,202 control subjects of European ancestry (see Methods; **S1 Fig**)[17]. Individuals were genotyped using the Illumina HumanHap550 or Quad610 Beadchip. Multi-dimensional scaling was used to infer ancestry, and the first twenty components were recorded for subsequent use as co-variates in association testing to control for potential population substructure. To generate imputed genotypes, we first selected SNPs present on both platforms that passed our quality control metrics and applied SHAPEIT to infer haplotypes[23]. We then utilized IMPUTE2 [24] with default parameters and Ne = 20000, along with a multi-population reference panel from the world-wide 1000 Genomes Project Phase 1 Release 3 to impute genotypes across the entire genome. For quality control purposes, variants with minor allele frequency (MAF) <1% and/or IMPUTE2-info quality score <0.7 were removed following imputation. The remaining variants were tested for association with neuroblastoma using the frequentist association test under the additive model using the “score” method implemented in SNPTEST [25] (**Fig 1 and S2 Fig**). The genomic inflation factor was 1.04 (**S3 Fig**).

### Refinement of known neuroblastoma susceptibility loci

**Fig 2:**
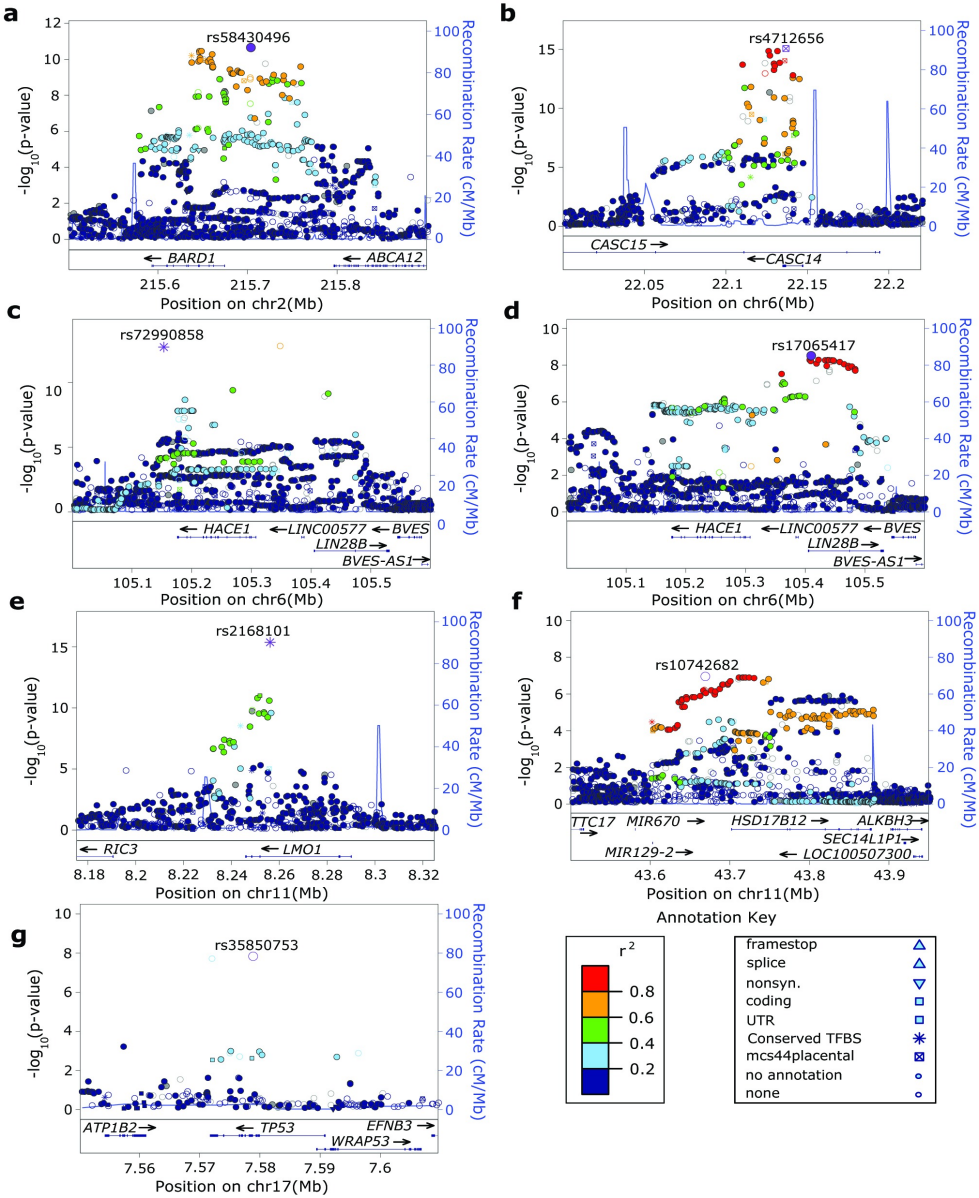
Genome-wide imputation confirms previously reported neuroblastoma susceptibility loci and identifies additional variants of greater statistical significance. Regional association plots of genotyped and imputed SNPs at previously reported neuroblastoma susceptibility loci identified by GWAS. Plots generated using LocusZoom. Y-axes represent the significance of association (-log10 transformed P values) and recombination rate. SNPs are color-coded based on pair-wise linkage disequilibrium (r^2^) with the most statistically significant SNP. **(a)** 2q35 locus implicating *BARD1* (rs58430496: p = 3.05 x 10–11; OR: 1.36, 95% CI: 1.25–1.48) **(b)** 6p22 locus implicating *CASC15* (rs4712656: p = 8.07 x 10–16; OR: 1.37, 95% CI: 1.27–1.47) **(c)** 6q16-q21 locus implicating *HACE1* (rs72990858: p = 1.37 x 10–13; OR: 0.59, 95% CI: 0.51–0.69) **(d)** independent association at 6q16-q21 after conditioning on rs72990858 implicates *LIN28B* (rs17065417: p = 4.72 x 10–9; OR: 0.70, 95% CI: 0.62–0.80) **(e)** 11p15 locus implicating *LMO1* (rs2168101: p = 3.18 x 10–16; OR: 0.70, 95% CI: 0.70–0.65) **(f)** 11p11 locus implicating *HSD17B12* (rs10742682: p = 1.31 x 10–7; OR: 1.24, 95% CI: 1.15–1.34) and (g) 17p13 implicating *TP53* (rs35850753: p = 1.38 x 10–8; OR: 1.95, 95% CI: 1.57–2.43).

We first confirmed previous reports of neuroblastoma-associated loci, and identified variants of greater statistical significance through imputation at each locus (**Fig 2; S2–S8 Tables**). Specifically, we observed association at 2q35 implicating *BARD1* [13] (**Fig 2A**, rs58430496: p = 3.05 x 10^−11^; OR: 1.36, 95% CI: 1.25–1.48), 6p22 implicating *CASC15* [11], (**Fig 2B,** rs4712656: p = 8.07 x 10^−16^; OR: 1.37, 95% CI: 1.27–1.47), and 6q16-q21 implicating *HACE1* [17] (**Fig 2C,** rs72990858: p = 1.37 x 10^−13^; OR: 0.59, 95% CI: 0.51–0.69). After conditioning on rs72990858 at 6q16, we identified a second independent association signal at 6q16-q21 implicating *LIN28B* [17] (**Fig 2D,** rs17065417: p = 4.72 x 10^−9^; OR: 0.70, 95% CI: 0.62–0.80). We also confirmed association at 11p15 implicating *LMO1* [15] (**Fig 2E,** rs2168101: p = 3.18 x 10^−16^; OR: 0.70, 95% CI: 0.70–0.65), 11p11 implicating *HSD17B12* [16] (**Fig 2F,** rs10742682: p = 1.31 x 10^−7^; OR: 1.24, 95% CI: 1.15–1.34) 17p13 implicating *TP53* [18] (**Fig 2G,** rs35850753: p = 1.39 x 10^−8^; OR: 1.95, 95% CI: 1.57–2.43).

### Discovery of new neuroblastoma susceptibility loci at 3q25 and 4p16

**Fig 3:**
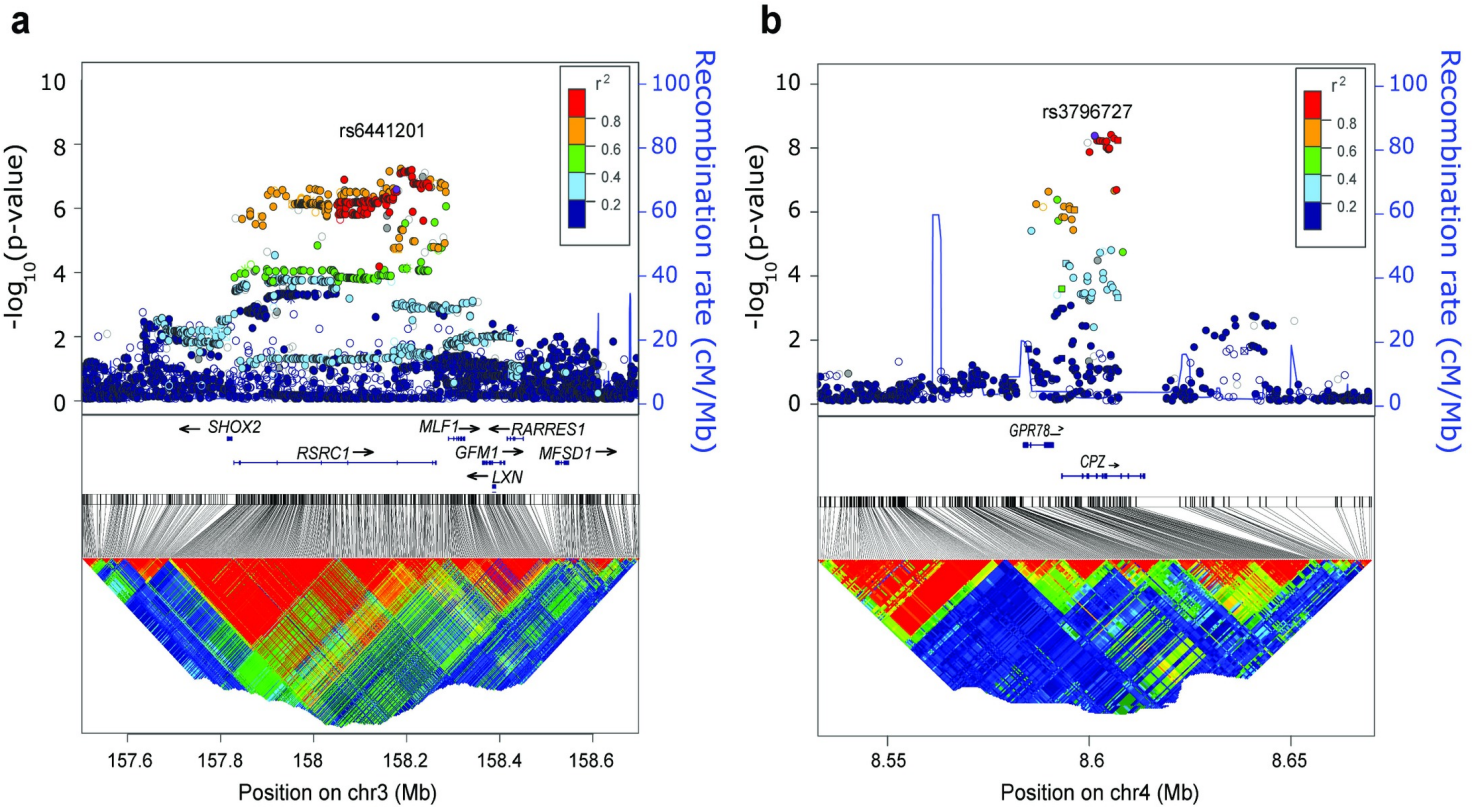
Discovery of neuroblastoma susceptibility loci at chromosome 3q25 and 4p16. Regional association plots of genotyped and imputed SNPs novel susceptibility loci. Plots were generated using LocusZoom. Y-axes represent the significance of association (-log10 transformed P values) and the recombination rate. SNPs are color-coded based on pair-wise linkage disequilibrium (r^2^) with indicated SNPs **(a)** 3q25 locus: rs6441201 shown in purple (3.01 x 10^−7^; Odds Ratio: 1.21, 95% C.I.: 1.12–1.30). **(b)** 4p16 locus: rs3796727 shown in purple (p = 5.25 x 10^−9^; Odds Ratio: 1.26, 95% C.I.: 1.16–1.36).

We observed two new genome-wide significant associations, the first at 3q25 (rs6441201: p = 3.01 x 10^−7^; Odds Ratio: 1.21, 95% C.I.: 1.12–1.30; **Fig 3A**; **Table 1**; **S9 Table**) and the other at 4p16 (rs3796727: p = 5.25 x 10^−9^; Odds Ratio: 1.26, 95% C.I.: 1.16–1.36; **Fig 3B**; **Table 1**; **S10 Table)**. The novel association signal at 3q25 spans a large 470-Kb linkage disequilibrium (LD) block in the HapMap CEU population, encompasses the arginine/serine-rich coiled-coil 1 (*RSRC1*) gene, and maps just upstream of the myeloid leukemia factor 1 gene (*MLF1*) (**Fig 3A**). The signal at 4p16 marks an approximate 27.5-Kb LD block in the CEU population and maps within the carboxypeptidase Z (*CPZ*) gene (**Fig 3B**).

**Table 1:** Statistically significant and replicated SNP associations at 3q25 and 4p16.

SNP	Major Allele	MinorAllele	Cohort^†^	Minor Allele Frequency Cases	Minor Allele Frequency Controls	P-value^‡^	OR^§^ (95% CI^║^)
rs6441201	G	A	European American	0.52 (n = 2,101)	0.47 (n = 4,202)	3.01 x 10^−7^	1.21 (1.12–1.30)
			African American	0.68 (n = 365)	0.64 (n = 2,491)	5.70 x 10^−3^	1.23 (1.04–1.45)
			United Kingdom	0.54 (n = 371)	0.47 (n = 1,122)	8.45 x 10^−4^	1.32 (1.12–1.56)
			Italian	0.50 (n = 427)	0.46 (n = 783)	0.11	1.15 (0.97–1.36)
			**Combined**			**1.21 x 10**^**−11**^	**1.23 (1.16–1.31)**
rs3796725	C	T	European American	0.35 (n = 2,101)	0.31 (n = 4,202)	1.41 x 10^−8^	1.23 (1.15–1.34)
			United Kingdom	0.33 (n = 353)	0.29 (n = 1,057)	0.028	1.23 (1.02–1.48)
			Italian	0.29 (n = 428)	0.23 (n = 759)	4.36 x 10^−3^	1.33 (1.10–1.61)
			**Combined**			**2.08 x 10**^**−11**^	**1.29 (1.19–1.38)**
rs3796727	G	A	European American	0.35 (n = 2,101)	0.30 (n = 4,202)	5.25 x 10^−9^	1.26 (1.16–1.36)
			United Kingdom	0.35 (n = 362)	0.29 (n = 1,094)	1.71 x 10^−3^	1.33 (1.11–1.59)
			Italian	0.31 (n = 432)	0.26 (n = 766)	0.010	1.28 (1.07–1.54)
			**Combined**			**1.26 x 10**^**−12**^	**1.30 (1.21–1.40)**

^**†**^ No deviations from Hardy-Weinberg equilibrium were observed (P>0.001) in all cohorts.

^‡ ^Allelic P-values; combined P-values from meta-analysis using METAL.

^§ ^OR: Odds Ratio with respect to the minor (risk) allele.

^║^CI: Confidence Interval

### Functional annotation of neuroblastoma-associated variants

To identify potential causal variants at each susceptibility locus, we developed an annotation tool incorporating data from ENCODE [26], the Roadmap Epigenomics Project [27], evolutionary conservation, and transcription factor binding motifs (see Methods). We applied this tool to all variants with a discovery p-value < 10^−4^, MAF > 0.005, and info score > 0.5. This approach confirmed the recently identified causal variant (rs2168101) at the *LMO1* locus shown to disrupt a canonical GATA binding site in neuroblastoma[20], and identified several other variants that warrant further study. (**S2–S10 Tables**).

### Conditional, interaction, and clinical correlative analyses

To investigate whether more than one association signal may exist at 3q25 or 4p16, we conditioned our analysis of 3q25 on rs6441201 and our analysis of 4p16 on rs3796727. No evidence for a separate association signal was observed at either locus (**S4 Fig**). In addition, no association was observed between rs6441201 or rs3796727 genotypes and clinical/biological covariates, including markers of tumor aggressiveness (**S11 and S12 Tables**). An interaction analysis between rs6441201 or rs3796727 and the most statistically significant SNPs at each of the previously reported susceptibility loci revealed only weak evidence for epistasis (**S13 Table**), suggesting that these loci may contribute independently to neuroblastoma risk.

### Replication of 3p25 and 4p16 association signals in three independent cohorts

We next sought to replicate the new 3q25 and 4p16 association signals in three independent cohorts (**S2 Fig**). First, we analyzed an African American cohort of 365 neuroblastoma cases and 2,491 genetically matched controls [28]. These individuals were genotyped on the Illumina HumanHap550 or Quad-610 bead chips, and SHAPEIT and IMPUTE2 [24] were applied to infer genotypes at the 3q25 and 4p16 loci using data from the 1000 Genomes Phase I Release 3 in a manner similar to the European American cohort. Utilizing the proportion of African admixture as a covariate to correct for varying degrees of admixture among our samples, we confirmed the association of rs6441201 at 3q25 (p = 5.70 x 10^−3^; Odds Ratio: 1.23, 95% CI: 1.04–1.45; **Table 1; S5 Fig**). Genotype imputation at the 4p16 locus was of low confidence in this cohort and therefore was not included. Next, we performed PCR-based genotyping in two additional independent cohorts for the top genotyped SNP at 3q25 (rs6441201), and two SNPs at the 4p16 locus since they were imputed (rs3796727 and rs3796725). First, we genotyped an Italian cohort of 427 neuroblastoma cases and 783 controls and observed a trend toward association in the same direction seen in the European and African American samples at 3q25 (rs6441201: P = 0.11, OR: 1.15, 95% CI: 0.97–1.36) and a robust replication at 4p16 (rs3796727: P = 0.010, OR: 1.28, 95% CI: 1.07–1.54; rs3796725: P = 4.36 x 10^−3^, OR: 1.33, 95% CI: 1.01–1.61 **Table 1**). Second, we genotyped both SNPs in a cohort of 371 cases and 1,122 controls from the United Kingdom, and confirmed all associations (rs6441201: P = 8.45 x 10^−4^, OR: 1.32, 95% CI: 1.12–1.56; rs3796727: P = 1.71 x 10^−3^, OR: 1.33, 95% CI: 1.11–1.59; rs3796725: P = 0.028, OR: 1.23 95% CI: 1.02–1.48; **Table 1**). Meta-analysis using the inverse-variance method within METAL[29] resulted in a highly significant associations with neuroblastoma (**Table 1**; rs6441201: P = 1.21x10^-11^, Odds Ratio 1.23, 95% CI:1.16–1.31 and rs3796727: P = 126x10^-12^, Odds Ratio 1.30, 95% CI:1.21–1.40; rs3796725: P = 2.08 x 10^−11^, Odds Ratio 1.29, 95% CI:1.19–1.38).

### rs3796727 genotype correlates with methylation status of *CPZ* at 4p16

**Fig 4:**
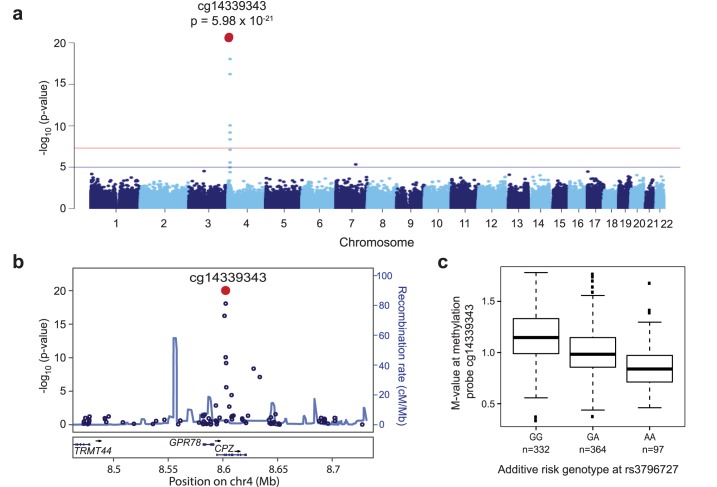
Methylation genome-wide association study (meGWAS) identifies rs3796727 as a methylation quantitative trait loci (meQTL) for sites in *CPZ*. (a) Manhattan plot of meGWAS results in 793 individuals based on additive rs3796727 genotype. A single genome-wide significant association is identified and the most statistically significant methylation probe is labeled (cg14339343). (b) LocusZoom plot at 4p16 locus reveals cg14339343 maps to the CPZ gene. Y-axes represent the significance of association (-log10 transformed P values) and the recombination rate. (c) Box plot of M-values based on rs3796727 genotype in 793 individuals. The rs3796727 risk allele “A” is associated with decreased methylation.

To investigate whether the neuroblastoma susceptibility variants may function as methylation quantitative trail loci (meQTL), we performed a methylation genome-wide association study based on additive risk genotype of rs6441201 (3q25) or rs3796727 (4p16) in a cohort of 769 individuals without cancer for whom we have both SNP and methylation array data, as described previously [30]. Briefly, M-values (log2 ratio between the methylated and unmethylated probe intensities [31]) were compared using an additive model based on SNP genotype. Principal component analysis (PCA) was first applied to infer ancestry (**S6 Fig**), and we focused initially on 395 individuals of European ancestry. No evidence was observed for rs6442101 functioning as a meQTL in this cohort. However, in our analysis of rs3796727 genotypes, we observed a single genome-wide significant meQTL signal mapping to the same neuroblastoma-associated locus at 4p16 (cg14339343, p = 1.33 x 10^−16^; **S7 Fig**; **S14 Table**); this signal replicated in the independent cohort comprised of 332 individuals of African ancestry (cg14339343, p = 1.36 x 10^−6^ **S8 Fig**; **S15 Table**). Analyzing all 769 individuals together in a multi-ethnic meGWAS yielded a highly significant association between rs3796727 genotype and methylation status of cg14339343 (cg14339343, p = 5.98 x 10^−21^ **Fig 4A**; **S16 Table**). Closer examination revealed that this meQTL resides directly within the 5′ UTR of the *CPZ* gene (**Fig 4B**), and the rs3796727 risk allele is associated with decreased methylation (**Fig 4C, S9 Fig**). These data suggest that rs3796727 genotype may influence *CPZ* expression. While RNA was not available to assess CPZ expression in these individuals, interrogation of the Genotype-Tissue Expression (GTEx) Portal revealed that *CPZ* expression was primarily limited to ovary, cervix and fallopian tube (**S10 Fig**). Cervix and fallopian tube did not include matched genotype data and thus eQTL analysis was not possible, but ovary tissue showed increased *CPZ* expression in cells homozygous for the rs3796727 risk allele (p = 0.17; **S11 Fig**). While not reaching statistical significance, this trend is consistent with the observed genotype-methylation correlation. Taken together, these data suggest that rs3796727 genotype may be associated with decreased methylation and increased *CPZ* expression; further study is necessary to confirm this role for rs3796727 in neuroblastoma directly.

### rs6441201 at 3q25 is a multi-tissue expression quantitative trait loci (eQTL)

To determine if the neuroblastoma-associated SNPs at 3q25 are eQTLs, we utilized the GTEx Portal. The rs6441201 variant at 3q25 was identified as a multi-tissue cis-eQTL for both *RSRC1* (p = 1.05 x 10^−78^; **S12 Fig**) and *LOC100996447*, a recently discovered long non-coding RNA located at 3q25 (p = 1.14 x 10^−145^; **S13 Fig**). In addition, rs6441201 was identified as a cis-eQTL for *MLF1* in esophagus (p = 6.33 x 10^−11^; **S14 Fig**).

### rs6441201 risk alleles are associated with increased MLF1 expression and *MLF1* silencing results in decreased cell growth in neuroblastoma cells

**Fig 5:**
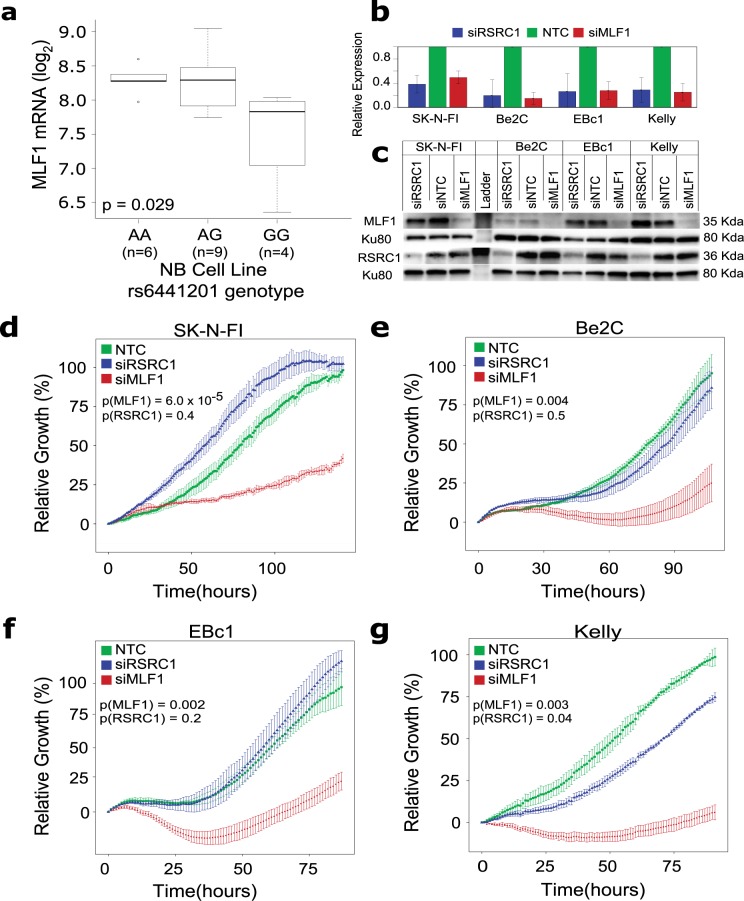
rs6441201 risk allele at 3q25 correlates with increased *MLF1* expression and *MLF1* silencing results in decreased cell growth in neuroblastoma cells. **(a)** *MLF1* mRNA expression is significantly higher in neuroblastoma cell lines harboring one or more copies of the rs6441201 risk allele (A) compared to neuroblastoma cell lines homozygous for the protective allele (GG). **(b)** Silencing of RSRC1 or MLF1 expression using pooled siRNAs resulted in 50–90% reduced mRNA levels by real-time quantitative PCR in neuroblastoma cell lines. **(c)** Confirmation by Western blot of knockdown at the protein level for RSRC1 and MLF1 after siRNA mediated silencing in neuroblastoma cell lines. **(d-g)** siRNA mediated silencing of MLF1 results in significant growth inhibition of neuroblastoma cells compared to non-targeting control siRNA; no effect was observed upon silencing of RSRC1. Cell growth measured by real-time cell sensing system (RT-ces).

We next analyzed a set of 19 neuroblastoma cell lines with matched genome-wide SNP genotyping and mRNA expression data. The rs6441201 variant was not observed to be an eQTL for *RSRC1* in neuroblastoma cells. However, *MLF1* expression was significantly higher in neuroblastoma cells harboring the rs6441201 risk allele compared those homozygous for the protective allele (*P* = 0.02; **Fig 5A**). We further interrogated seven additional genes in the region, but did not observe association of rs6441201 genotype with mRNA levels. Consistent with these findings, silencing of *MLF1*, but not *RSRC1*, using pooled siRNA resulted in significant cell growth inhibition in neuroblastoma cells (**Fig 5A–5D**).

## Discussion

Neuroblastoma is an embryonal tumor of the autonomic nervous system thought to arise from developing and incompletely committed precursor cells derived from neural crest tissues; it is the most common cancer diagnosed in the first year of life [1]. Here, in order to identify germline genetic risk factors and genes influencing neuroblastoma tumorigenesis, we performed a genome-wide association studying (GWAS) comprising a total of 3,264 neuroblastoma patients and 8,598 healthy control subjects from four independent cohorts. Two new neuroblastoma susceptibility loci were identified, one at chromosome 3q25 and the other at 4p16. The 4p16 variants map to the *CPZ* gene locus, and the 3q25 variants map within *RSRC1* and upstream of *MLF1*.

The *CPZ* gene encodes a member of the carboxypeptidase E subfamily of metallocarboxypeptidases which represent Zn-dependent enzymes implicated in intra- and extracellular processing of proteins. Through an unbiased meGWAS, we observed strong evidence for rs3796727 functioning as a meQTL for sites within the 5′ UTR of *CPZ*. Specifically, the rs3796727 risk allele was associated with decreased methylation, suggesting the risk allele may be associated with increased expression of *CPZ*. CPZ is a Zn-dependent enzyme with an N-terminal cysteine-rich domain (CRD) and a C-terminal catalytic domain. CPZ is enriched in the extracellular matrix and expressed during early embryogenesis. In addition to containing a metallocarboxypeptidase domain, CPZ also contains a Cys-rich domain with homology to Wnt-binding proteins [32]. Indeed, studies in chick embryos suggest that CPZ is involved in WNT signaling[33]. In addition, *CPZ* has been shown to modulate Wnt/beta-catenin signaling and terminal differentiation of growth plate chondrocytes[34]. Among the tissues interrogated in GTEx, *CPZ* expression was primarily observed in ovary, where there was a trend toward increased expression in cells homozygous for the risk allele (**S10 and S11 Figs**). Our methylation GWAS based on additive risk allele at the 4p16 susceptibility locus revealed significantly decreased methylation in the 5' UTR of CPZ of cells harboring the risk allele, consistent with increased CPZ expression. Matched RNA was not available to assess mRNA expression in the methylation GWAS cohort, and a genotype-expression correlation was not observed in neuroblastoma cell lines. However, CPZ may influence tumor initiation and thus require assessment of precursor cells from the developing sympathetic nervous system.

The 3q25 variants map within *RSRC1* which encodes a member of the serine and arginine rich-related protein family. The gene product has been shown to play a role in constitutive and alternative splicing, and is involved in the recognition of the 3′ splice site during the second step of splicing [35]. Variants in *RSRC1* are associated with the neurological disease schizophrenia, and RSRC1 is involved in prenatal brain development and cell migration to forebrain structures [36]. *RSRC2*, a member of the same gene family, has been proposed as a tumor suppressor gene in esophageal carcinogenesis[37]. Increased expression of *RSRC2* has been observed in neuroblastomas harboring somatic gain of chromosome 12q [38], and a *MIER2*-*RSRC1* fusion has been observed in prostate cancer [39]. Taken together, existing studies suggest that *RSRC1* may play an important role in both neural stem cell proliferation and cancer development.

The *MLF1* gene, also mapped to 3q25, encodes an oncoprotein that is thought to play a role in the phenotypic determination of hematopoetic cells. It was first identified as the C-terminal partner of the leukemic fusion protein nucleophosmin (NPM)-MLF1 that resulted from a t(3;5)(q25.1;q34) chromosomal translocation [40]. *MLF1* is overexpressed in more than 25% of MDS-associated cases of AML, in the malignant transformation phase of MDS, and in lung squamous cell carcinoma [41, 42]. *MLF1* overexpression is thought to suppress a rise in the CDK inhibitor *CDKN1B*, preventing the activation of Epo-activated terminal differentiation pathway and promoting proliferation [43]. *MLF1* is expressed in a wide variety of tissues, shuttles between the cytoplasm and the nucleus, and has also been shown to reduce proliferation by stabilizing the activity of TP53 by suppressing its E3 ubiquitin ligase, COP1 [44]. These data suggest that *MLF1* may play both a tumor suppressing and an oncogenic role depending on the biological context.

Since both *RSRC1* and *MLF1* have been previously implicated in cancer, we investigated the 3q25 locus in more detail. Based on GTEx data, rs6441201 is a multi-tissue eQTL for both *RSRC1* and a recently discovered long non-coding RNA *LOC100996447* at 3q25. While we did not observe a genotype-expression correlation for *RSRC1* or *LOC100996447* in neuroblastoma cells, we cannot rule out the possibility that variants at 3q25 influence expression of *RSRC1* and/or *LOC100996447* genes early in tumorigenesis within developing neural crest cells. However, *MLF1* expression was observed in nineteen distinct neuroblastoma cell lines interrogated in this study, with the highest expression in cells homozygous for the risk allele at rs6441201. Silencing of MLF1 resulted in significant growth inhibition in four distinct neuroblastoma cell lines. Taken together, these data are consistent with the hypothesis that *MLF1* promotes neuroblastoma tumorigenesis, and that the 3q25 risk alleles are associated with growth advantage through increased *MLF1* expression. Given that the observed cell growth phenotype was independent of rs6441201 genotype, alternative mechanisms driving MLF1 expression to promote neuroblastoma cell growth likely exist.

In conclusion, here we refine previously reported susceptibility loci, identify common variation at chromosome 3q25 and 4p16 associated with neuroblastoma, and provide insight into potential causal variants at the newly identified susceptibility loci. The newly associated variants at 4p16 are located within *CPZ*, and the top associated SNP is a meQTL for sites located directly within the 5′ UTR of *CPZ*. The associated variants at 3q25 appear to function in *cis* to alter *MLF1* expression in neuroblastoma. Based on initial functional studies, it is likely that germline susceptibility alleles at 3q25 play and important role in both initiation and disease progression. Ongoing studies will further elucidate the role of both *CPZ and MLF1* in neuroblastoma tumorigenesis.

## Materials and methods

### Genotype imputation and association testing

A primary European-American cohort of 2,101 cases and 4,202 matched controls were assayed with Illumina HumanHap550 v1, Illumina HumanHap550 v3, and Illumina Human610 SNP arrays as previously described [17]. Genotypes were phased using SHAPEIT [23] v2.r790 and data from 1000 Genomes Phase 1 Release 3. Subsequently, imputation was performed genome-wide using IMPUTE2 [24] v2.3.1 for all SNPs and indel variants annotated in 1000 Genomes Phase I Release 3. To minimize potential errors in phasing and imputation performed genome-wide, we employed a genome-tiling approach. Each position in the genome was covered by a minimum of three tiles (sliding windows). Variants with MAF <1% and/or IMPUTE2-info quality score <0.7 were removed. Testing for association with neuroblastoma was performed under an additive genetic effect model using the frequentist likelihood score method implemented in SNPTEST [25] v2.4.1. After genome-wide assessment, regions with p < 5.0 x 10^−7^ were re-imputed without tiling and tested for association in a similar manner. Genotypes for a previously described African-American replication cohort of 365 cases 2491 controls [28] were imputed and tested for neuroblastoma association using the same analytic pipeline. Statistical adjustment for gender was performed in both cohorts. For population stratification adjustment, the first 20 multidimensional scaling (MDS) components were included as covariates in the European-American cohort, while a measure of African admixture as estimated by the ADMIXTURE software program was used in the African-American cohort.

### Replication in Italian and United Kingdom cohorts

Genotyping of the top associated SNPs at *MLF1* (rs6441201) and *RSRC1* (rs3796725 and rs3796727) was performed using TaqMan SNP genotyping assays (Life Technology). The Italian cohort was comprised of a total of 432 neuroblastoma cases and 780 controls. The replication cohort from the United Kingdom included 371 cases and 1,122 controls in total. Association with neuroblastoma was assessed using an additive genetic effect model of the frequentist likelihood score method implemented in SNPTEST [25] v2.4.1 in the same manner as the discovery cohort.

### Genotype imputation and methylation association testing

DNA from 769 children without cancer was extracted from blood and genotyped using Illumina HumanHap550 v1, Illumina HumanHap550 v3, and Illumina Human610 SNP arrays. DNA from the same individuals was also profiled for genome-wide methylation using Illumina 450K methylation arrays. Genotypes were phased using SHAPEIT [23] v2.r790 and data from 1000 Genomes Phase 1 Release 3. Subsequently, imputation was performed genome-wide using IMPUTE2 [24] v2.3.1 for all SNPs and indel variants annotated in 1000 Genomes Phase I Release 3. Principal component analysis (PCA) was performed based on genotype data and ancestry was inferred. A threshold of 0.9 was applied to rs3796727 imputed genotype probabilities for the purpose of methylation association testing; genotypes from individuals not reaching this threshold were excluded. Association testing was subsequently performed using linear regression with the R software.

### Meta-analysis

Meta-analysis was performed using the inverse-variance method within the METAL [29] software package, and a fixed-effects model was assumed.

### Methylation data analyses

Genome-wide methylation profiles were generated from gDNA isolated from peripheral blood mononuclear cells from a total of 854 subjects recruited by the Center for Applied Genomics (CAG) at the Children’s Hospital of Philadelphia (CHOP) on the Infinium HumanMethylation450 BeadChip Kit according to the manufacturers' protocols. and analyzed as Methylation data were exported from GenomeStudio and subjected to quantile color balance adjustment, background level correction, and simple scaling normalization as described previously [30]. Principle component analysis identified 425 subjects of European ancestry, 374 African Americans, 20 East Asians, and 24 Hispanics among these subjects. Methylation probes known to overlap with common SNPs, were identified and removed using the IMA R package. M-values (the log2 ratio between the methylated and unmethylated probe intensities) were extracted and stored as a matrix. Additive genotypes at rs3796727 for subjects of European ancestry were extracted from existing genotyping data using PLINK. There are a total of 402 subjects of European ancestry without missing genotype at rs3796727 and extreme outlier values of methylation M-values (≥median M-value of the genotype group±3 s.d.). Methylation data in gene *CPZ* were analyzed as the response variable in a linear regression, with genotype at as the predictor variable among these 402 subjects. Sex, age, and 10 genotype-derived principle components were included as covariates. Linear regression and generation of boxplots was performed using base packages in R.

### Genome-wide mRNA expression profiling of neuroblastoma cell lines

Genome-wide mRNA expression profiling in neuroblastoma cell lines was performed using the Illumina WG-6 expression array according to the manufacturer’s specifications. Data were normalized using the average normalization method provided in Illumina GenomeStudio software. ANOVA test was performed at the gene level to assess differential expression in cell lines. *P* < 0.05 was considered significant. Data is available from the Gene Expression Omnibus (GEO) database (Accession: GSE78061).

### RT-PCR in neuroblastoma cell lines

TaqMan Gene expression assays for *MLF1* (Hs00963682_m1), *RSRC1* (HS00963694_m1) and *HPRT* (Hs02800695_m1) were purchased through Life Technologies. Reactions were set up in triplicate. Starting with 200 ng RNA, reverse transcription was performed followed by 1:4 dilution and 2 ul of cDNA was subsequently used in a 10-μl reaction with 1× TaqMan Universal PCR Master Mix (Life Technologies). Standard curves were generated using serial dilutions of cDNA from the neuroblastoma cell line Kelly, produced in the same RT reaction as the experimental samples. Samples were amplified on an Applied Biosystems 7900HT Sequence Detection System using standard cycling conditions, and data were collected and analyzed with SDS 2.3 software. *MLF1* and *RSRC1* expression levels were normalized to *HPRT* expression.

### MLF1 and RSRC1 protein detection

Neuroblastoma cell lines were grown in T75 flasks under standard cell culture conditions. Cells were plated into 6 well plates for transfection with siRNA, 2 wells per target for protein analysis. Replicate samples were pooled on collection. Whole-cell lysates were extracted with 100 μl of protein lysis buffer containing Tris Base (25mM), NaCl (150 mM), EGTA and EDTA (1 mM each), NaF (10 mM) DTT (1 mM), Triton X-100 (1%), and protease/phosphatase inhibitors (Cell Signaling, #5872) on ice for at least 30 minutes before brief sonication. After 15 min of centrifugation at 4°C, the supernatant was removed, and protein quantification was performed using the Pierce BCA Protein Assay Kit (Life Technologies, 23225). Lysates (12 μg) were separated on 10% Criterion TGX gels (BioRad) and were transferred to PVDF membranes. Membranes were washed and incubated with antibodies directed against MLF1 (Abcam, ab70211), RSRC1 (Abcam, ab106650) and Ku80 (Cell Signaling, 2753). All blocking and antibody dilution was performed in 5% milk in TBST.

### *MLF1 and RSRC1* knockdown and monitoring of cell growth

For routine maintenance, cells were grown in RPMI 1640 complete medium (Gibco, 22400) containing 10% FBS (Hyclone, SH 30073–03), 1× antibiotic antimycotic (Gibco, 15240–062) and 2 mM l-glutamine (Gibco, 25030). On day 0, cells were seeded in triplicate into antibiotic-free medium in 96-well RT-CES plates (ACEA). On day 1, using DharmaFECT 1 (Dharmacon, T-2001-03, 0.1%), cells were transiently transfected with 25 nM of either a non-targeting negative control siRNA (Dharmacon, D-00810-10-20) or pooled siRNA directed against *MLF1* (L-019478-00-0005) or *RSRC1* (L-028584-01-0005). Real-time cell growth was monitored every hour for at least 96 h using the RT-CES system, as previously described. Data presented are representative of at least three independent experiments. To monitor efficiency of *MLF1 and RSRC1* knockdown, transfection was performed as described, and RNA was isolated 48 hours later using the Qiagen mini extraction kit. Total RNA (200 ng) was primed with oligo(dT) and reverse transcribed using SuperScript First Strand Synthesis System for RT-PCR (Life Technologies). Quantitative RT-PCR using TaqMan gene expression assays (ABI) was performed as described above. Similarly, protein was isolated 72 hours after transfection to monitor MLF1 and RSRC1 protein knockdown using Western blot analysis as described.

### GWAS annotation tool

Variants directly genotyped, or imputed from the 1000 Genomics phase 1 release 3 data with discovery p-value < 10^−4^, MAF > 0.005, and info score > 0.5 were annotated and ranked based on a DNase I hypersensitivity data, evolutionary conservation, transcription factor binding site scores, and Roadmap Epigenomics data. Conservation scores were computed as the average of the phastCons46way Placental UCSC conservation track score for all bases from the −10 position to the +10 position surrounding each candidate variant. A DNase I hypersensitivity score was calculated by counting the number of sequencing tags from the −100 position to the +100 position around each candidate variant in ENCODE data for the neuroblastoma cell line, SK-N-SH. Scanning for transcription factor binding motifs was performed using a custom implementation of the MATCH algorithm[45] using JASPAR 2014[46] position weight matrices (PWMs) as input. Briefly, to quantify the conservation of position *i* in a PWM described by a frequency matrix, *f*_*i,B*_, the information vector was computed as follows:

$$I(i)=\sum_{B\in (A,C,G,T)}f_{i,B}\log_{2}\left(4f_{i,B}\right)$$

For a given input sequence, *b*_*i*_, an absolute information-weighted match score was computed as

$$Score=\sum_{i=1}^{L}I(i)f_{i,b_{i}}$$

and a normalized matrix similarity score (mSS) was computed as previously described.

This scan was completed both for the entire human reference genome (hg19) and a modified version of the reference genome (hg19_alt), where each reference base was replaced by its alternative base at each SNP position. A match was called for a PWM if the mSS was greater than 0.8 for either hg19 or hg19_alt at a given position overlapping a SNP. At these positions, an mSS difference (delta-nrm) and an absolute score difference (delta-abs) were computed between hg19_alt and hg19 as two separate metrics to quantify the predicted effect of each SNP on transcription factor binding.

## Web resources

The URLs for data presented herein are as follows:

1000 Genomes Project, http://www.1000genomes.org

LiftOver, http://genome.ucsc.edu/cgi-bin/hgLiftOver

SHAPEIT, https://mathgen.stats.ox.ac.uk/genetics_software/shapeit/shapeit

IMPUTE2, http://mathgen.stats.ox.ac.uk/impute/impute_v2

SNPTEST, https://mathgen.stats.ox.ac.uk/genetics_software/snptest/snptest

LocusZoom, http://csg.sph.umich.edu/locuszoom

METAL, http://www.sph.umich.edu/csg/abecasis/metal

## Supporting information

S1 Table Neuroblastoma patient characteristics. (PDF)

S2 Table SNPTEST results at 2q35 NB susceptibility locus in European American Discovery Cohort (2,101 cases; 4,202 controls). (XLSX)

S3 Table SNPTEST results at 6p22 NB susceptibility locus in European American Discovery Cohort (2,101 cases; 4,202 controls). (XLSX)

S4 Table SNPTEST results at 6q16-q21 NB susceptibility locus in European American Discovery Cohort (2,101 cases; 4,202 controls). (XLSX)

S5 Table SNPTEST results at 6q16-q21 NB susceptibility locus conditioned on rs72990858 in European American Discovery Cohort (2,101 cases; 4,202 controls). (XLSX)

S6 Table SNPTEST results at 11p15 NB susceptibility locus in European American Discovery Cohort (2,101 cases; 4,202 controls). (XLSX)

S7 Table SNPTEST results at 11p11 NB susceptibility locus in European American Discovery Cohort (2,101 cases; 4,202 controls). (XLSX)

S8 Table SNPTEST results at 17p13 NB susceptibility locus in European American Discovery Cohort (2,101 cases; 4,202 controls). (XLSX)

S9 Table SNPTEST results at novel 3q25 NB susceptibility locus in European American Discovery Cohort (2,101 cases; 4,202 controls). (XLSX)

S10 Table SNPTEST results at novel 4p16 NB susceptibility locus in European American Discovery Cohort (2,101 cases; 4,202 controls). (XLSX)

S11 Table Correlation of rs6442101 genotype with clinical variables. (PDF)

S12 Table Correlation of rs3796727 genotype with clinical variables. (PDF)

S13 Table Epistasis analysis results. (PDF)

S14 Table European American methylation GWAS results at 4p16 locus based on additive rs3796727 genotype. (XLSX)

S15 Table African American methylation GWAS results at 4p16 locus based on additive rs3796727 genotype. (XLSX)

S16 Table Combined European and African American methylation GWAS results at 4p16 locus based on additive rs3796727 genotype. (XLSX)

S1 Fig MDS plot of discovery and replication cohorts. a. European-ancestry discovery cohort. b. African American replication cohort.
(PDF)

S2 Fig Flow diagram of discovery and replication efforts. Shown are the Discovery and Replication cohorts utilized in this study along with ancestry information and the number of variants tested. Two novel loci were replicated, including a single genotyped variant from 3q25 (rs6442101) and two variants from 4p16 (rs3796725 and rs3796727). Variants located at 4p16 were not imputed in Replication Cohort #1 (African American) with acceptable quality, and therefore were not considered. These variants, along with rs6442101 at 3q25, were directly genotyped using a PCR-based approach in Replication cohorts #2 and #3.
(PDF)

S3 Fig QQ plot of discovery GWAS. Plotted are the expected vs. observed–log_10_ p-values from the European ancestry discovery cohort. Genomic inflation factor was 1.04.
(PDF)

S4 Fig Conditional association results. Genomic position based on hg19. **a.** conditioned on rs6442101. The original signal is completely ablated, and a putative second signal of modest statistical significance is observed downstream of *MLF1*. **b.** conditioned on rs3796727. SNPs mapping to the 4p16 susceptibility locus are no longer statistically significant indicating a single association signal.
(PDF)

S5 Fig LocusZoom plot of 3q25 locus in African American replication cohort. Regional association plot of genotyped and imputed SNPs at 3q25 locus. Y-axes represent the significance of association (-log10 transformed P values) and the recombination rate. SNPs are color-coded based on pair-wise linkage disequilibrium (r^2^) with indicated SNPs at q25 locus: rs6441201 shown in purple (p = 5.70 x 10^−3^; Odds Ratio: 1.23, 95% CI: 1.04–1.45).
(PDF)

S6 Fig PCA of individuals considered for methylation GWAS. Green: European ancestry. Black: African ancestry. Red: Asian ancestry.
(PDF)

S7 Fig Manhattan plot of European American methylation GWAS. Association of rs3796727 with cg14339343 methylation status is confirmed when restricting to individuals of European ancestry (p = 1.33 x 10^−16^). See S14 Table for detailed methylation GWAS results at the 4p16 locus.
(PDF)

S8 Fig Manhattan plot of African American methylation GWAS. Association of rs3796727 with cg14339343 methylation status is confirmed when restricting to individuals of African ancestry (p = 1.36 x 10^−6^). See S15 Table for detailed methylation GWAS results at the 4p16 locus.
(PDF)

S9 Fig Risk allele at rs3796727 is associated with decreased methylation at *CPZ*. M-value for cg14339343, located in 5′ UTR of CPZ, is plotted based on additive rs3796727 risk allele (0,1,or 2 alleles). **(a)** Plot restricted to children of European ancestry. **(b)** Plot restricted to children of African American ancestry.
(PDF)

S10 Fig *CPZ* expression across normal tissues in GTEx. *CPZ* exhibits tissue specific expression. *CPZ* is primarily expressed in Ovary. CPZ is also expressed in mammary tissue, cervix (ecto and endo), mucosa in esophagus, fallopian tube, and vagina. Minimal or no expression is observed in remaining tissues profiled.
(PDF)

S11 Fig Expression of *CPZ* in ovarian tissue. Expression of *CPZ* is higher in ovarian tissue homozygous for the rs3796727 neuroblastoma-associated risk allele at 4p16, though this did not reach statistical significance (p = 0.17). Data and figure from GTEx portal (Analysis Release V6).
(PDF)

S12 Fig rs6441201 is a multi-tissue eQTL for *RSRC1*. Expression of RSRC1 is significantly correlated with rs6441201 genotype. Data and figure from GTEx portal (Analysis Release V6).
(PDF)

S13 Fig rs6441201 is a multi-tissue eQTL for LOC100996447. Expression of LOC100996447 (RP11-538P18.2), a long non-coding RNA, is significantly correlated with rs6441201 genotype. Data and figure from GTEx portal (Analysis Release V6).
(PDF)

S14 Fig rs6441201 is an eQTL for *MLF1* in esophagus. Expression of *MLF1* is significantly correlated with rs6441201 genotype in esophagus mucosa (p **=** 6.3 x 10^**−**11^). Data and figure from GTEx portal (Analysis Release V6).
(PDF)
