# Supplementary material for: Common variants upstream of MLF1 at 3q25 and within CPZ at 4p16 associated with neuroblastoma
Source: PLoS Genet. 2017 May 18;13(5):e1006787. doi: 10.1371/journal.pgen.1006787 (PMC5456408; doi:10.1371/journal.pgen.1006787)
Supplement: S1 Table — (PDF) [file pgen.1006787.s001.pdf]

Table S1. Neuroblastoma patient characteristics.

Characteristic	Discovery§ (European Ancestry) Blood DNA n=2,101	Replication§ (African American) Blood DNA n=365
**Age**		
< 1 yr	736 (36)	99 (27)
≥ 1 yr	1,324 (64)	263 (73)
Not Available	41	3
**INSS Stage**		
Stage 1	388 (20)	49 (14)
Stage 2	272 (14)	47 (13)
Stage 3	327 (16)	61 (18)
Stage 4	875 (44)	172 (49)
Stage 4S	123 (6)	22 (6)
Not Available	116	14
MYCN		
Not Amplified	1,566 (82)	278 (84)
Amplified	342 (18)	54 (16)
Not available	193	33
**Histology**		
Favorable	932 (56)	139 (49)
Unfavorable	735 (44)	144 (51)
Not available	434	82
**DNA index**		
Hyperdiploid	616 (66)	63 (61)
Diploid	316 (34)	46 (39)
Not available	1169	246
**Risk**		
Low	705 (36)	98 (29)
Intermediate	386 (20)	63 (19)
High	853 (44)	176 (52)
Not available	157	28

§ Frequency within each clinical/biological subset is listed in parentheses.
