# Supplementary material for: Common variants upstream of MLF1 at 3q25 and within CPZ at 4p16 associated with neuroblastoma
Source: PLoS Genet. 2017 May 18;13(5):e1006787. doi: 10.1371/journal.pgen.1006787 (PMC5456408; doi:10.1371/journal.pgen.1006787)
Supplement: S11 Table — (PDF) [file pgen.1006787.s011.pdf]

Table S11. Correlation of rs6442101 genotype with clinical variables.

				P-value [2]	
	**AA[1]**	**GA[1]**	**GG[1]**	**AA vs. GG**	**AA/AG vs. GG**
**Stage 4**	246 (27%)	413 (48%)	200 (25%)	0.3092	0.4550
**Not Stage 4**	290 (29%)	525 (48%)	269 (23%)		
***MYCN* Amp**	96 (29%)	156 (47%)	82 (24%)	0.0587	0.0140
***MYCN* Not Amp**	414 (30%)	718 (52%)	256 (18%)		
**High risk**	234 (28%)	402 (48%)	200 (24%)	0.9489	1.000
**Not High risk**	299 (28%)	522 (48%)	258 (24%)		
**Diploid**	159 (28%)	279 (48%)	140 (24%)	0.7775	0.8584
**Hyperdiploid**	333 (28%)	569 (48%)	281 (24%)		
**Unfavorable Histology**	218 (29%)	338 (46%)	183 (25%)	0.7339	0.7742
**Favorable Histology**	252 (27%)	449 (49%)	223 (24%)		
**Age >= 18 months**	287 (27%)	499 (48%)	236 (25%)	0.4492	0.3405
**Age < 18 months**	256 (28%)	445 (49%)	233 (23%)		

1: The AA, GA and GG genotypes represent homozygous risk genotype, heterozygous risk genotype and homozygous non-risk (protective) genotype, respectively.
2: Two-sided Fisher's exact test
