# Supplementary material for: Common variants upstream of MLF1 at 3q25 and within CPZ at 4p16 associated with neuroblastoma
Source: PLoS Genet. 2017 May 18;13(5):e1006787. doi: 10.1371/journal.pgen.1006787 (PMC5456408; doi:10.1371/journal.pgen.1006787)
Supplement: S12 Table — (PDF) [file pgen.1006787.s012.pdf]

Table S12. Correlation of rs3796727 genotype with clinical variables.

				P-value [2]	
	AA[1]	GA[1]	GG[1]	AA vs. GG	AA/AG vs. GG
**Stage 4**	81 (11%)	343 (42%)	342 (46%)	0.7467	0.5609
**Not Stage 4**	113 (12%)	413 (42%)	451 (46%)		
***MYCN* Amp**	26 (9%)	120 (41%)	147 (50%)	0.1351	0.1367
***MYCN* Not Amp**	152 (11%)	583 (43%)	606 (45%)		
**High risk**	77 (10%)	335 (45%)	332 (45%)	0.8052	0.3796
**Not High risk**	111 (11%)	407 (42%)	455 (47%)		
**Diploid**	54 (10%)	217 (42%)	246 (48%)	0.5904	0.4845
**Hyperdiploid**	118 (11%)	460 (43%)	484 (46%)		
**Unfavorable Histology**	68 (10%)	279 (43%)	307 (47%)	0.6597	0.6012
**Favorable Histology**	92 (11%)	367 (44%)	383 (45%)		
**Age >= 18 months**	99 (11%)	384 (45%)	426 (44%)	0.5235	0.2499
**Age < 18 months**	96 (11%)	377 (42%)	373 (47%)		

1: The AA, GA and GG genotypes represent homozygous risk genotype, heterozygous risk genotype and homozygous non-risk (protective) genotype, respectively.
2: Two-sided Fisher's exact test
