# Supplementary material for: Common variants upstream of MLF1 at 3q25 and within CPZ at 4p16 associated with neuroblastoma
Source: PLoS Genet. 2017 May 18;13(5):e1006787. doi: 10.1371/journal.pgen.1006787 (PMC5456408; doi:10.1371/journal.pgen.1006787)
Supplement: S13 Table — (PDF) [file pgen.1006787.s013.pdf]

Table S13. Epistasis analysis results.

SNP1	SNP2	OR_INT	STAT	P
rs182632338	rs35850753	1.381	0.075	0.784
rs182632338	rs7952320	0.928	0.054	0.816
rs182632338	rs3796727	1.427	1.320	0.251
rs182632338	rs114532410	0.981	0.001	0.978
rs182632338	rs4712656	0.903	0.140	0.708
rs182632338	chr11:43708557:D	0.897	0.143	0.705
rs182632338	rs4945714	0.848	0.058	0.809
rs182632338	rs6787172	1.568	2.548	0.111
rs182632338	rs6932801	0.457	1.105	0.293
rs182632338	rs2070096	1.295	0.578	0.447
rs35850753	rs7952320	1.111	0.307	0.580
rs35850753	rs3796727	1.308	1.662	0.197
rs35850753	rs114532410	0.550	1.557	0.212
rs35850753	rs4712656	1.345	2.183	0.140
rs35850753	chr11:43708557:D	0.850	0.788	0.375
rs35850753	rs4945714	0.429	2.604	0.107
rs35850753	rs6787172	1.028	0.022	0.881
rs35850753	rs6932801	0.698	0.324	0.569
rs35850753	rs2070096	0.854	0.545	0.461
rs7952320	rs3796727	1.137	3.967	0.046
rs7952320	rs114532410	0.931	0.290	0.590
rs7952320	rs4712656	0.940	1.164	0.281
rs7952320	chr11:43708557:D	0.951	0.734	0.392
rs7952320	rs4945714	0.902	0.512	0.474
rs7952320	rs6787172	1.023	0.158	0.691
rs7952320	rs6932801	1.333	2.864	0.091
rs7952320	rs2070096	1.013	0.042	0.838
rs3796727	rs114532410	0.891	0.552	0.458
rs3796727	rs4712656	0.936	1.070	0.301
rs3796727	chr11:43708557:D	0.913	2.042	0.153
rs3796727	rs4945714	0.882	0.677	0.411
rs3796727	rs6787172	0.997	0.003	0.960
rs3796727	rs6932801	1.034	0.029	0.866
rs3796727	rs2070096	0.913	1.655	0.198
rs114532410	rs4712656	0.910	0.504	0.478
rs114532410	chr11:43708557:D	0.956	0.108	0.743
rs114532410	rs4945714	1.212	0.403	0.525
rs114532410	rs6787172	0.881	0.887	0.346
rs114532410	rs6932801	1.163	0.166	0.684
rs114532410	rs2070096	0.938	0.187	0.665
rs4712656	chr11:43708557:D	0.976	0.177	0.674
rs4712656	rs4945714	1.047	0.113	0.737
rs4712656	rs6787172	0.952	0.768	0.381
rs4712656	rs6932801	1.432	3.936	0.047
rs4712656	rs2070096	1.115	3.017	0.082
chr11:43708557:D	rs4945714	1.090	0.391	0.532
chr11:43708557:D	rs6787172	0.942	1.092	0.296
chr11:43708557:D	rs6932801	1.521	4.966	0.026
chr11:43708557:D	rs2070096	1.045	0.481	0.488
rs4945714	rs6787172	0.938	0.220	0.639
rs4945714	rs6932801	1.440	0.807	0.369
rs4945714	rs2070096	0.981	0.015	0.903
rs6787172	rs6932801	0.960	0.054	0.817
rs6787172	rs2070096	0.956	0.512	0.474
rs6932801	rs2070096	0.928	0.150	0.699
