# Supplementary figures and images for: Common variants upstream of MLF1 at 3q25 and within CPZ at 4p16 associated with neuroblastoma
Source: PLoS Genet. 2017 May 18;13(5):e1006787. doi: 10.1371/journal.pgen.1006787 (PMC5456408; doi:10.1371/journal.pgen.1006787)

**a**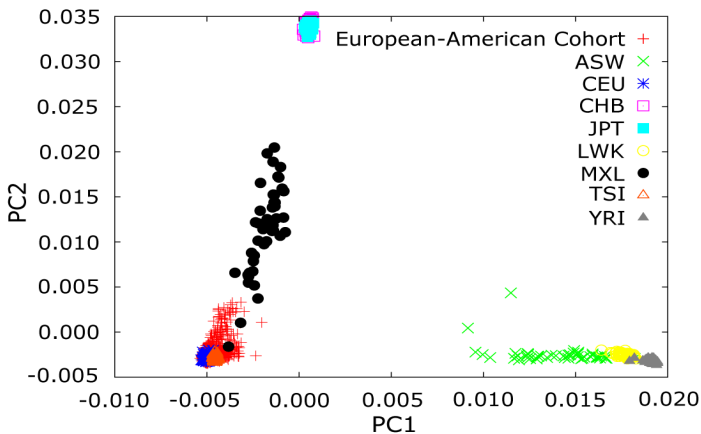**b**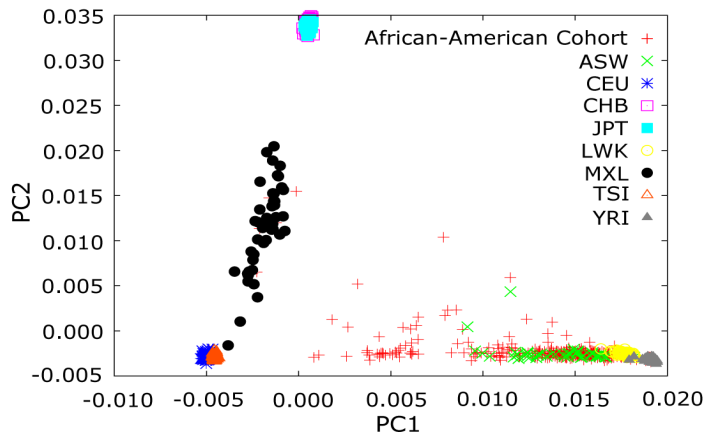

Supplement: S1 Fig — a. European-ancestry discovery cohort. b. African American replication cohort. (PDF) [file pgen.1006787.s017.pdf]

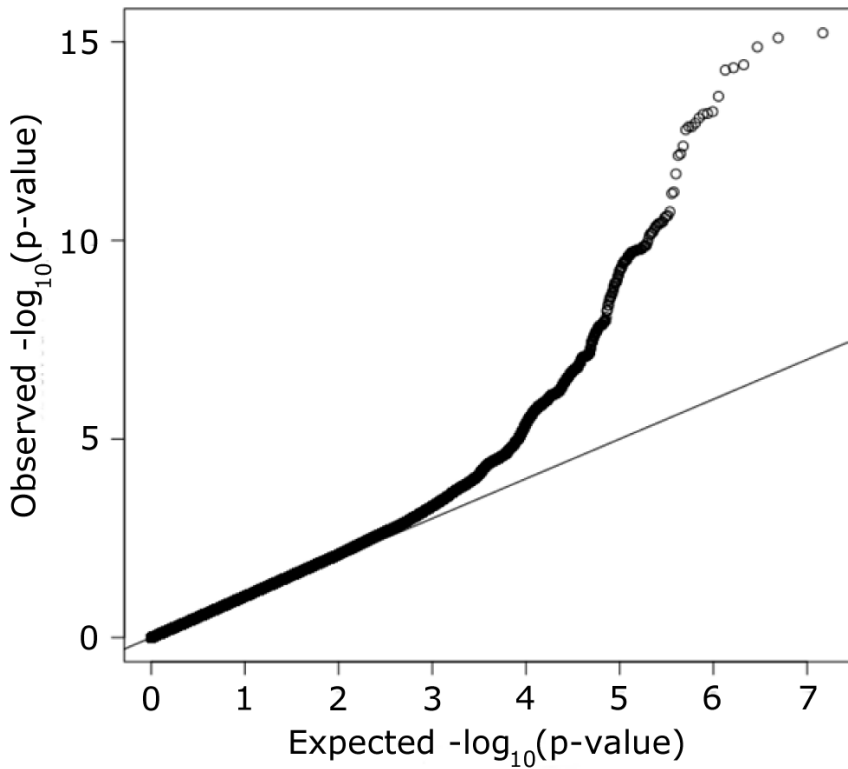

Supplement: S3 Fig — Plotted are the expected vs. observed–log10 p-values from the European ancestry discovery cohort. Genomic inflation factor was 1.04. (PDF) [file pgen.1006787.s019.pdf]

**a.**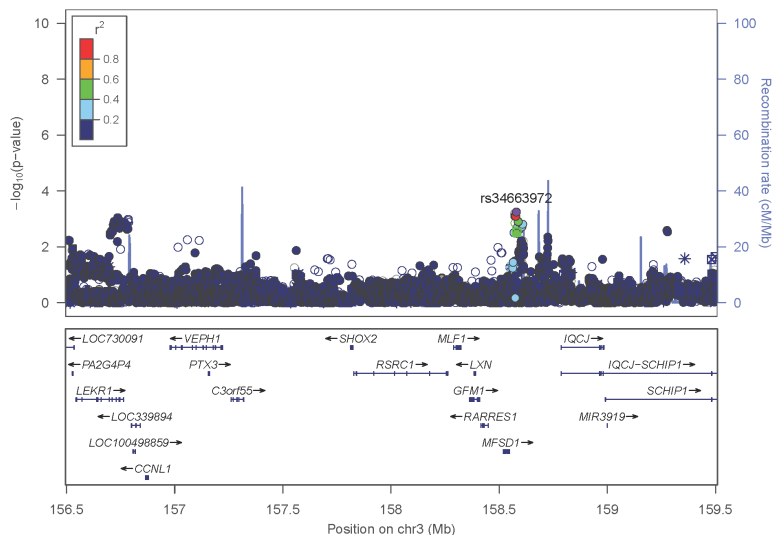**b.**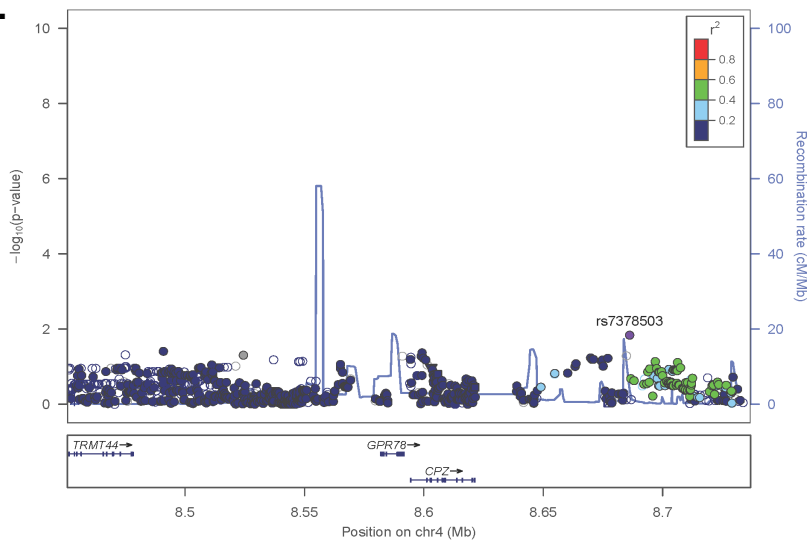

Supplement: S4 Fig — Genomic position based on hg19. a. conditioned on rs6442101. The original signal is completely ablated, and a putative second signal of modest statistical significance is observed downstream of MLF1. b. conditioned on rs3796727. SNPs mapping to the 4p16 susceptibility locus are no longer statistically significant indicating a single association signal. (PDF) [file pgen.1006787.s020.pdf]

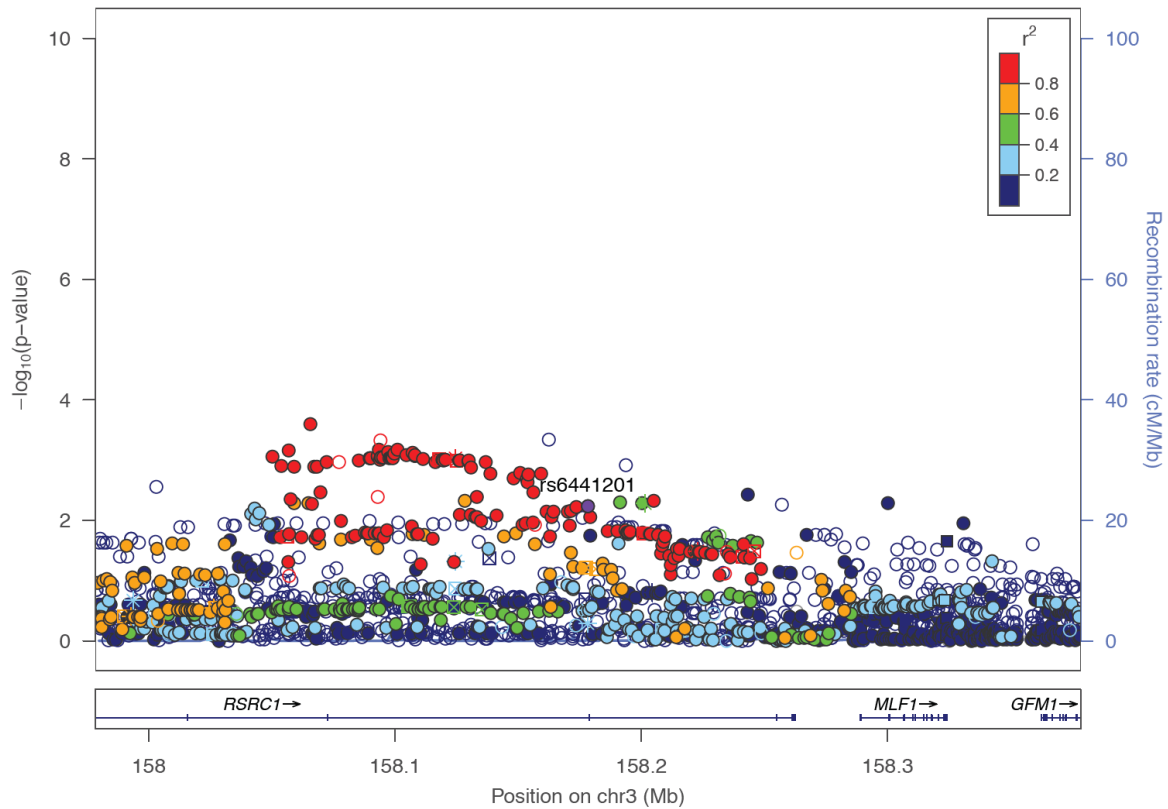

Supplement: S5 Fig — Regional association plot of genotyped and imputed SNPs at 3q25 locus. Y-axes represent the significance of association (-log10 transformed P values) and the recombination rate. SNPs are color-coded based on pair-wise linkage disequilibrium (r2) with indicated SNPs at q25 locus: rs6441201 shown in purple (p = 5.70 x 10^−3^; Odds Ratio: 1.23, 95% CI: 1.04–1.45). (PDF) [file pgen.1006787.s021.pdf]

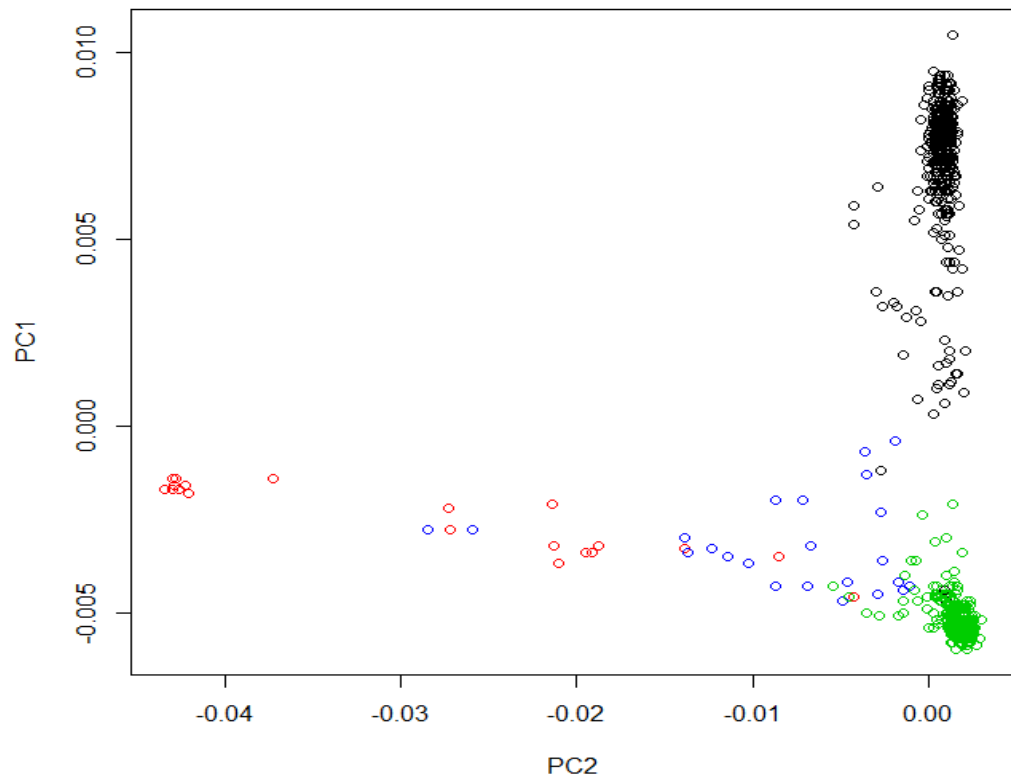

Supplement: S6 Fig — Green: European ancestry. Black: African ancestry. Red: Asian ancestry. (PDF) [file pgen.1006787.s022.pdf]

cg14339343

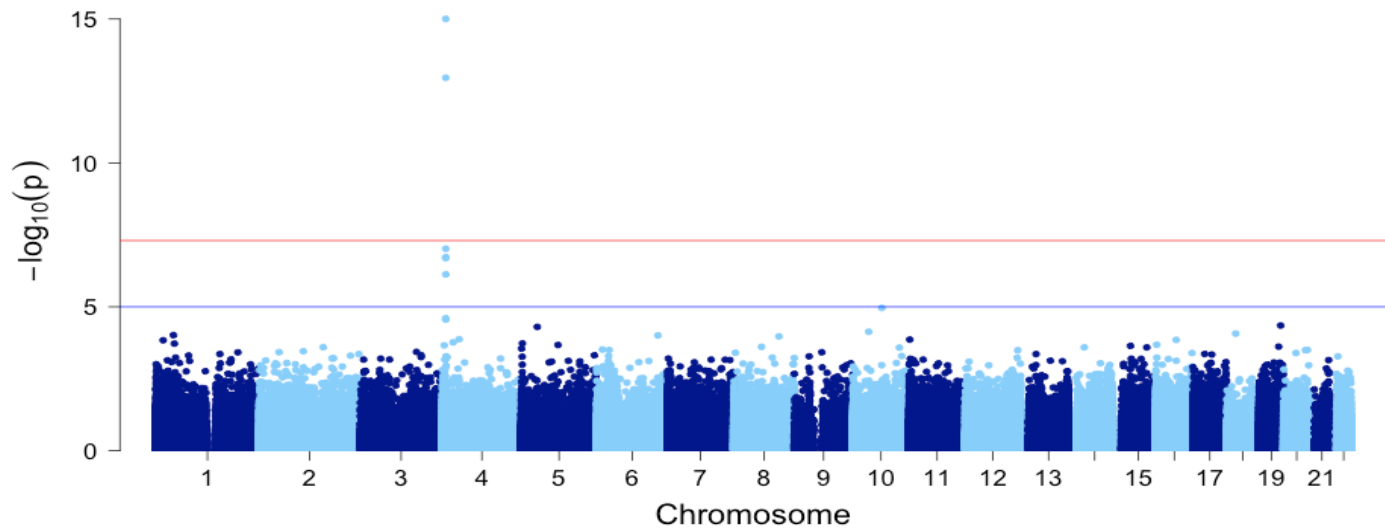

Supplement: S7 Fig — Association of rs3796727 with cg14339343 methylation status is confirmed when restricting to individuals of European ancestry (p = 1.33 x 10^−16^). See S14 Table for detailed methylation GWAS results at the 4p16 locus. (PDF) [file pgen.1006787.s023.pdf]

cg14339343

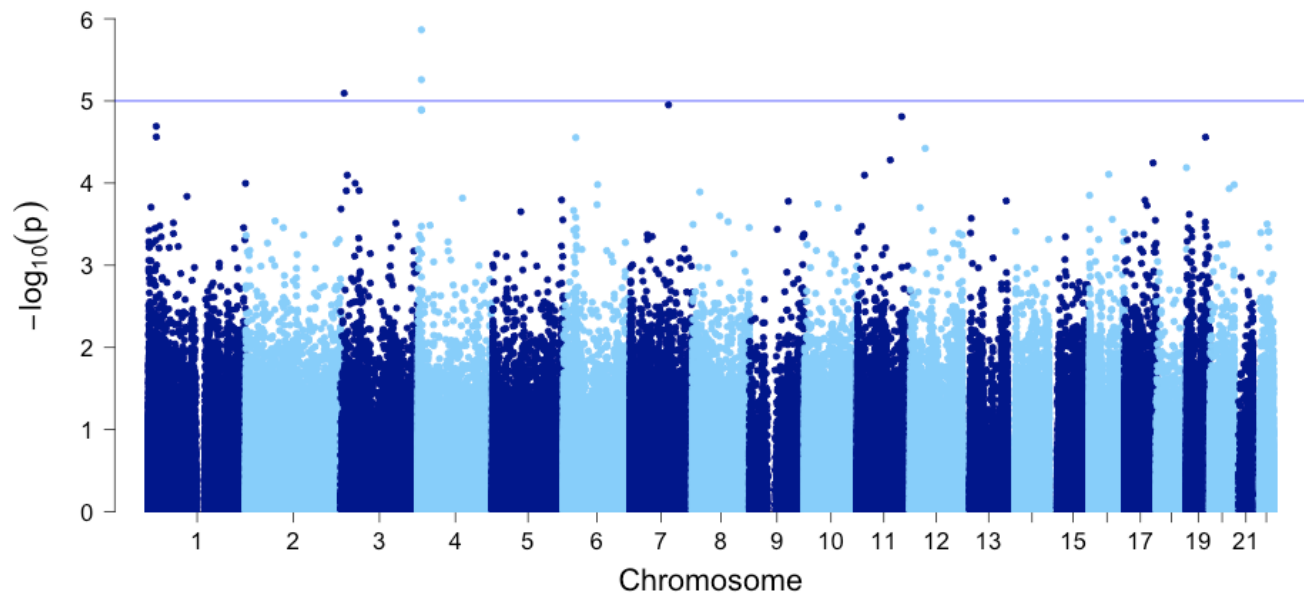

Supplement: S8 Fig — Association of rs3796727 with cg14339343 methylation status is confirmed when restricting to individuals of African ancestry (p = 1.36 x 10^−6^). See S15 Table for detailed methylation GWAS results at the 4p16 locus. (PDF) [file pgen.1006787.s024.pdf]

**a.**

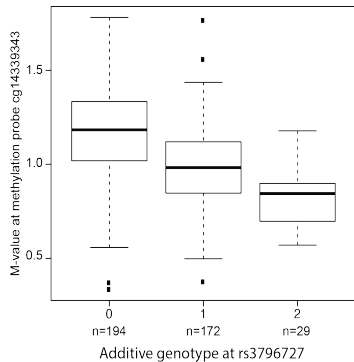

**b.**

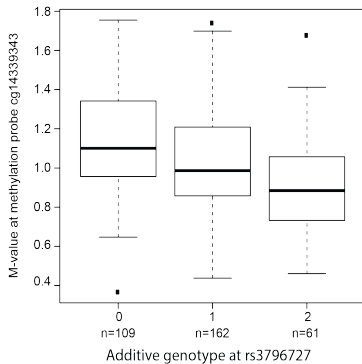

Supplement: S9 Fig — M-value for cg14339343, located in 5′ UTR of CPZ, is plotted based on additive rs3796727 risk allele (0,1,or 2 alleles). (a) Plot restricted to children of European ancestry. (b) Plot restricted to children of African American ancestry. (PDF) [file pgen.1006787.s025.pdf]

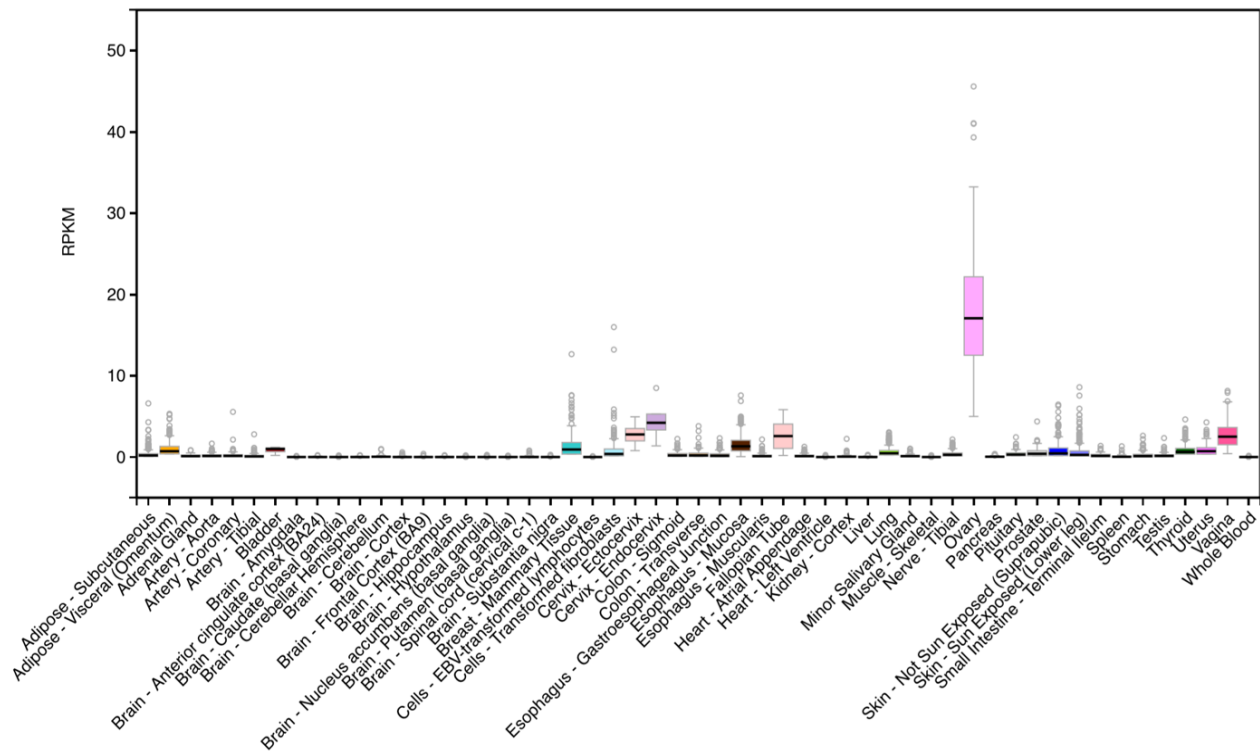

Supplement: S10 Fig — CPZ exhibits tissue specific expression. CPZ is primarily expressed in Ovary. CPZ is also expressed in mammary tissue, cervix (ecto and endo), mucosa in esophagus, fallopian tube, and vagina. Minimal or no expression is observed in remaining tissues profiled. (PDF) [file pgen.1006787.s026.pdf]

Ovary eQTL rs3796727 ENSG00000109625.14

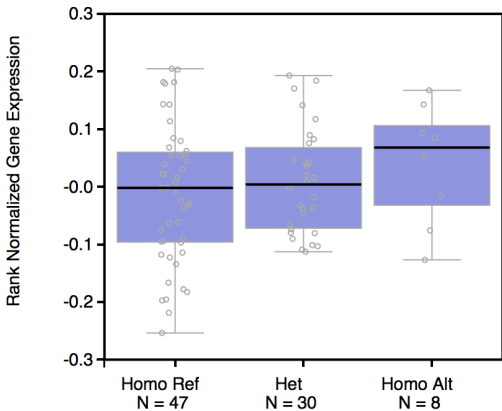

Supplement: S11 Fig — Expression of CPZ is higher in ovarian tissue homozygous for the rs3796727 neuroblastoma-associated risk allele at 4p16, though this did not reach statistical significance (p = 0.17). Data and figure from GTEx portal (Analysis Release V6). (PDF) [file pgen.1006787.s027.pdf]

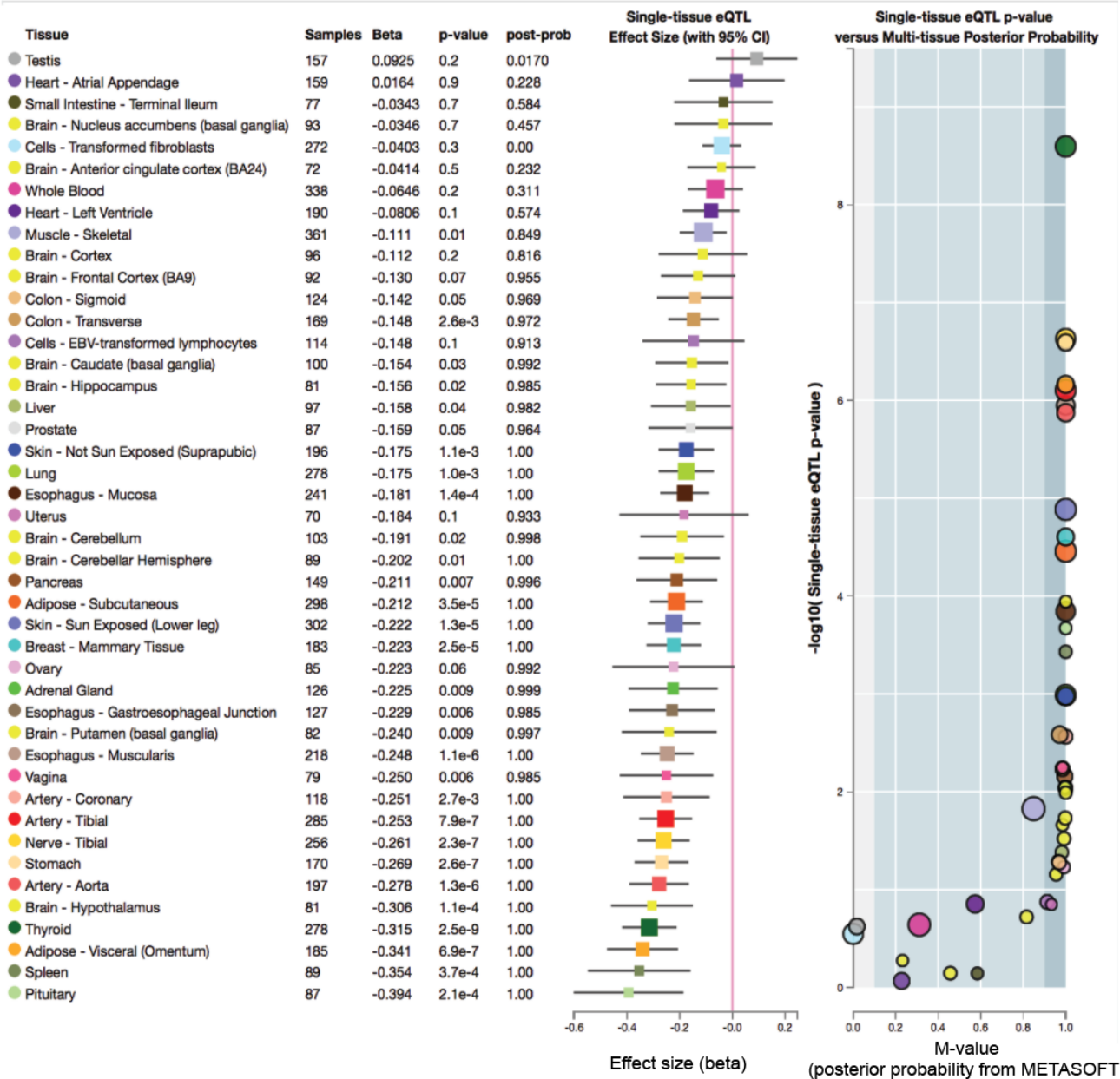

Supplement: S12 Fig — Expression of RSRC1 is significantly correlated with rs6441201 genotype. Data and figure from GTEx portal (Analysis Release V6). (PDF) [file pgen.1006787.s028.pdf]

Multi-tissue eQTL Comparison

ENSG00000243150.1 RP11-538P18.2 and rs6441201 eQTL (Meta Analysis RE2 P-Value: 1.14482e-145)

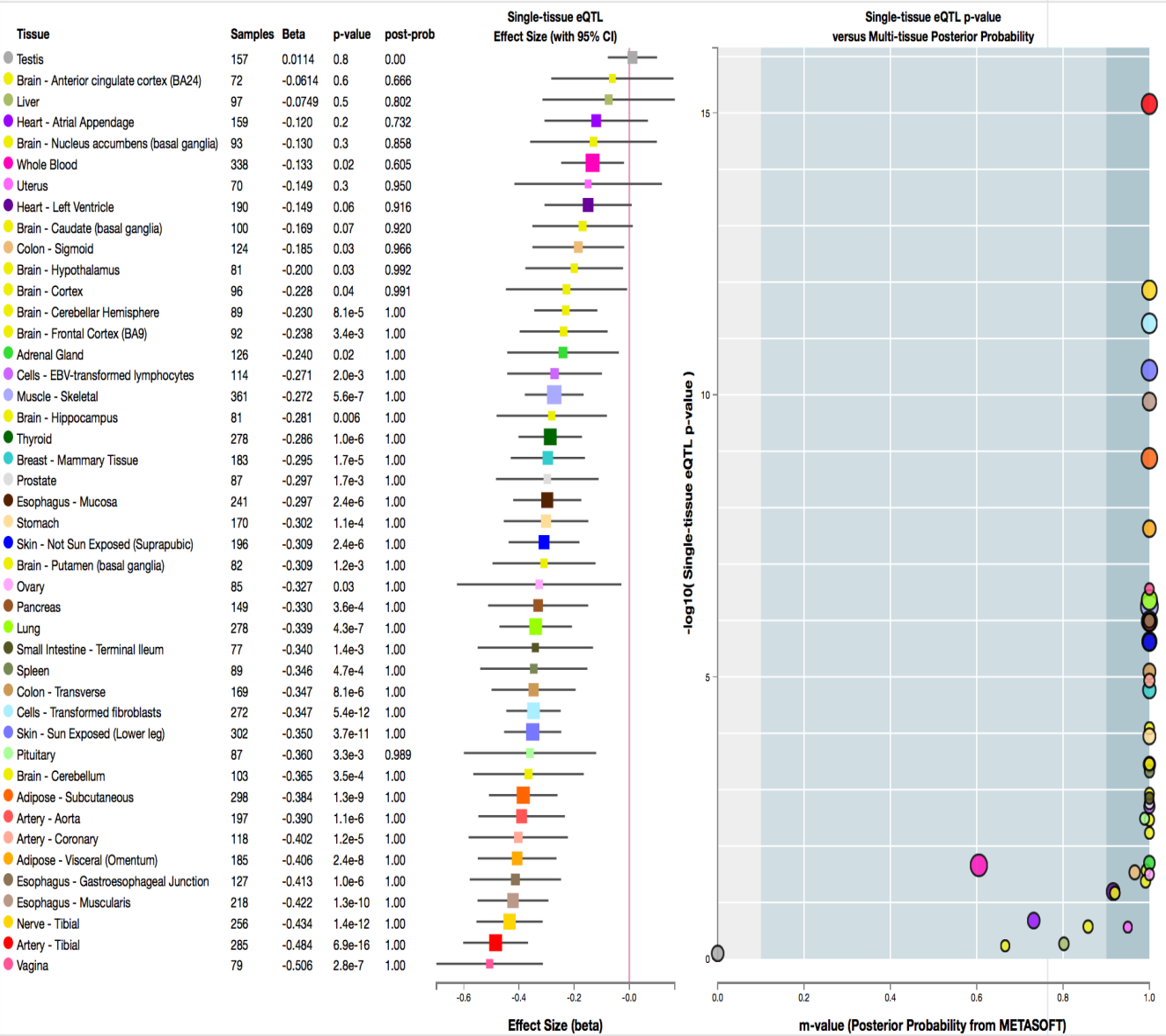

Supplement: S13 Fig — Expression of LOC100996447 (RP11-538P18.2), a long non-coding RNA, is significantly correlated with rs6441201 genotype. Data and figure from GTEx portal (Analysis Release V6). (PDF) [file pgen.1006787.s029.pdf]

Esophagus\_Mucosa eQTL rs6441201 ENSG00000178053.13

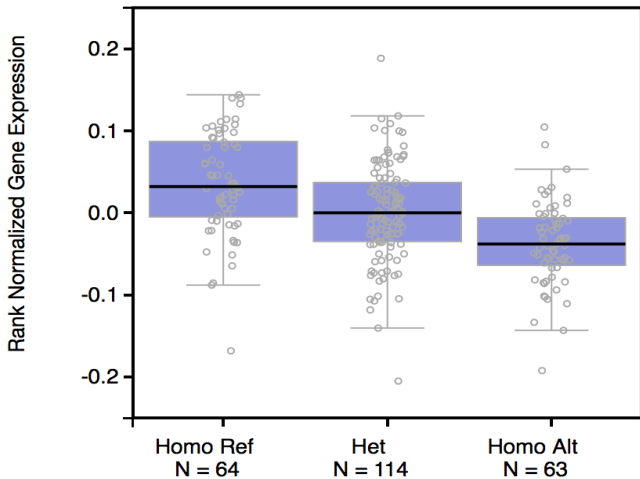

Supplement: S14 Fig — Expression of MLF1 is significantly correlated with rs6441201 genotype in esophagus mucosa (p = 6.3 x 10^−11^). Data and figure from GTEx portal (Analysis Release V6). (PDF) [file pgen.1006787.s030.pdf]
