# Supplementary material for: Common variants upstream of MLF1 at 3q25 and within CPZ at 4p16 associated with neuroblastoma
Source: PLoS Genet. 2017 May 18;13(5):e1006787. doi: 10.1371/journal.pgen.1006787 (PMC5456408; doi:10.1371/journal.pgen.1006787)
Supplement: S2 Fig — Shown are the Discovery and Replication cohorts utilized in this study along with ancestry information and the number of variants tested. Two novel loci were replicated, including a single genotyped variant from 3q25 (rs6442101) and two variants from 4p16 (rs3796725 and rs3796727). Variants located at 4p16 were not imputed in Replication Cohort #1 (African American) with acceptable quality, and therefore were not considered. These variants, along with rs6442101 at 3q25, were directly genotyped using a PCR-based approach in Replication cohorts #2 and #3. (PDF) [file pgen.1006787.s018.pdf]

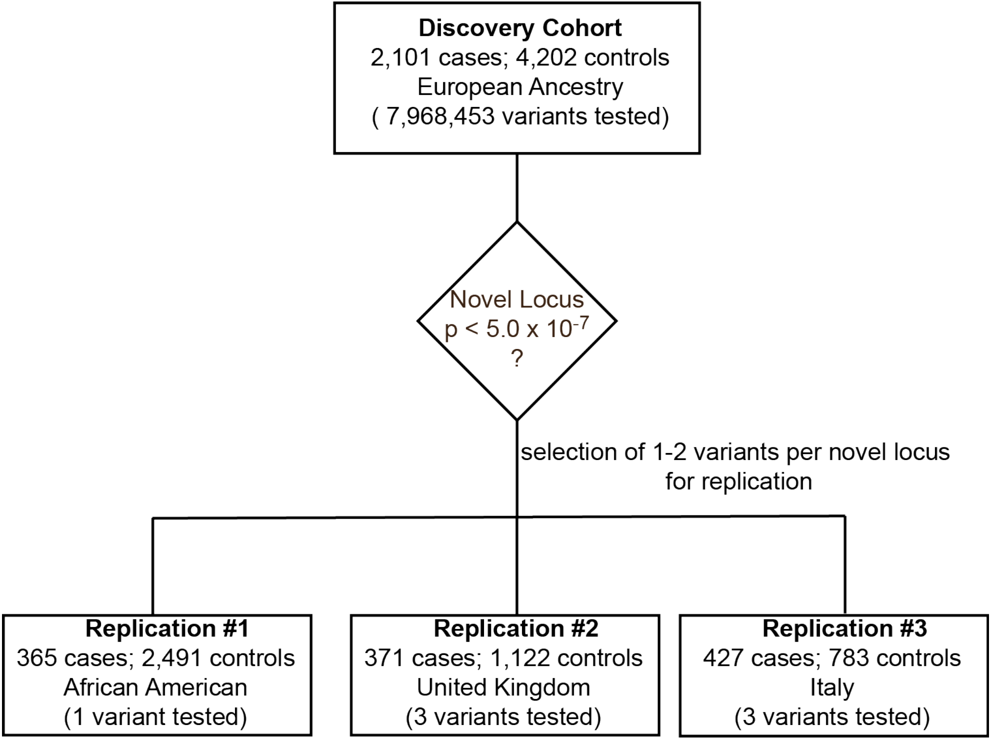
Discovery Cohort
2,101 cases; 4,202 controls
European Ancestry
(7,968,453 variants tested)
Novel Locus
p < 5.0 x 10-7
?
selection of 1-2 variants per novel locus
for replication
Replication #1
365 cases; 2,491 controls
African American
(1 variant tested)
Replication #2
371 cases; 1,122 controls
United Kingdom
(3 variants tested)
Replication #3
427 cases; 783 controls
Italy
(3 variants tested)
